# RETRACTION: Dietary Supplementation of the Antioxidant Curcumin Halts Systemic LPS‐Induced Neuroinflammation‐Associated Neurodegeneration and Memory/Synaptic Impairment via the JNK/NF‐*κ*B/Akt Signaling Pathway in Adult Rats

**DOI:** 10.1155/omcl/9837507

**Published:** 2026-06-23

**Authors:** Oxidative Medicine and Cellular Longevity

RETRACTION: M.S. Khan, T. Muhammad, M. Ikram, and M.O. Kim, “Dietary Supplementation of the Antioxidant Curcumin Halts Systemic LPS‐Induced Neuroinflammation‐Associated Neurodegeneration and Memory/Synaptic Impairment via the JNK/NF‐*κ*B/Akt Signaling Pathway in Adult Rats,” *Oxidative Medicine and Cellular Longevity* (2019): e7860650, https://doi.org/10.1155/2019/7860650.

The above article, published online on 7 November 2019 in Wiley Online Library (wileyonlinelibrary.com), has been retracted by agreement between the Journal Editor‐in‐Chief, Jeannette Vasquez Vivar; and Wiley Periodicals LLC. Concerns were raised by Elizabeth Bik on PubPeer [1] that the LPS and LPS+Cur panels in Figure 1G look similar, particularly the DAPI blue signals; and that the TNF‐α panel in Figure 2A appears identical to the Cyt c panel of Figure 3A. Additionally, issues were raised regarding Figure 2C and Figure 2J. The publisher confirmed these concerns that Figure 2C and 2J were later used by some of these authors without attribution [2, 3]. The authors responded by providing their raw image data, but their responses to the concerns regarding image duplication were insufficient. Because the images appear to be manipulated, the Editors have lost confidence in the results published. As a result, the data and conclusions are considered unreliable. Therefore, the article must be retracted.
